# Book Reviews

**Published:** 1893-11

**Authors:** 


### *BOOK REVIEWS.*

---

A DICTIONARY OF MEDICAL SCIENCE. By Robley Dunglison, M. D., LL. D., Late Professor of the Institutes of Medicine and Medical Jurisprudence in Jefferson Medical College of Philadelphia. Twenty-first edition, thoroughly revised and greatly enlarged, by Richard J. Dunglison, A. M., M. D. Lea Bros. & Co., Philadelphia.

There is no greater classic in medical literature than Dunglison's Dictionary. For more than thirty years it has been the standard. The edition of 1874 had about lived out its usefulness on account of the numerous changes and additions that have been made in recent years to our medical terminology. Hence the present twenty-first edition is very acceptable. Some improvements have been made in the old work which render it more useful both to the student and the physician. To the edition of 1874 there have been added forty-four thousand new terms and subjects. *Pronunciation* and *accentuation* of terms are now introduced for the first time. The *derivation* of words is also added, which will be found of great assistance. Some very excellent tables are incorporated in the body of the book under such headings as *Bacteria, Doses, Examination of Urine, Localizations of Lesions,* etc. This is the day of medical dictionaries. Some of them do not contain enough, and some contain too much. We think this one fulfils better than any other the requirements of a practical and serviceable dictionary. The medical profession we believe will receive the revised edition with genuine pleasure, and we doubt not it will continue to enjoy its well-merited position in the library of nearly every physician.

---

GRAY'S ANATOMY. A New American from the thirteenth English Edition. Philadelphia, Lea Brothers & Co., 1893.

The name of this book is too well known, and its character as a standard work too well established to need especial notice or praise. The publishers say each of the thirteen editions have been scrutinized by the foremost anatomists in order that the work might be kept up with the progress and knowledge of anatomy. In this addition there has been a thorough revision of the text, and where necessary for clearness the plates have been re-engraved.

Special stress is laid upon the fact that this Anatomy is intended for the practical use of students and practitioners, who must apply their anatomical knowledge to the practice of surgery.

The descriptions are made of each structure separately and not regionally, though the "relations" are always given.

There is quite a thorough description of the minute anatomy of the general structure of the body at the beginning of the book, and the illustrations are excellent.

Following the histological descriptions is a chapter on development, from the ovum to the matured fetus. "Descriptive and Surgical Anatomy" follows. The colored lines (red), indicating points of attachment of muscle to bone, are of great assistance to the student, as well as to one wishing to refresh his memory in a very few minutes. In muscular anatomy the arteries, veins and nerves related are shown in red, blue and yellow, respectively, in the plates. In the description of the arteries, veins and nerves, the plates show this same coloring of these structures; the lymphatics are not colored. New plates appear, showing dissections not shown in older editions.

The anatomical descriptions, as well as the "surgical anatomy," are full and complete. Here and there is a slight change in the name or a more particular description of a structure. M. B. H.

A TREATISE ON OPHTHALMOLOGY. By Adolph Alt, M. D. J. H. Chambers & Co., St. Louis.

This is the second edition of this work by Dr. Alt, and as such it has been somewhat enlarged from its original publication. While the work is not one of reference, it yet meets the aim as set forth by the author and as such will be welcomed by the profession. This aim of the author is succinctly expressed in the preface when he says "I have tried to adhere closely to the original plan, to make it a useful guide to the *general practitioner* who may be forced to take care of certain diseases." His illustrations are apt and good, and will no doubt be favorably received by the practitioners at large.

D. R.

---

THE PHARMACOPEIA OF THE UNITED STATES OF AMERICA. Seventh Decennial Revision, by the authority of the National Convention for revising the Pharmacopeia, held in Washington, A. D. 1890. Official from January 1, 1894. Published by the Committee of Revision. J. B. Lippincott & Co. Agents: P. Blakiston, Son & Co. 1893.

This edition of the United States Pharmacopeia is a handsomely printed book of over six hundred pages, being more than one hundred more than was found in the edition of 1880.

We note with regret that the Georgia Medical Association failed to send delegates to the convention, but were glad to see that our State was represented by Drs. G. F. Payne, H. R. Slack and R. H. Land.

The changes, while entirely too numerous to be noted in the limits of our space, are for the best. Ninety articles have been dropped and eighty-eight added, leaving in all nine hundred and ninety four medicines that are *official.* Among the most important additionsare acetanilid, aloin, caffeine, citrate, cocaine hydrochlorate, cascara sagrada and corn silk. None we think will regret the dropping of the eleven abstracts or the four plasters, but we are not so sure about inspissated ox gall. No patented or copyrighted medicines are recognized by this authority. Adopting the metric system *in toto* is certainly a great improvement on the last impracticable method of parts by weight, and is a long stride in the direction of scientific progress. Another change on the same line is in the chemical nomenclature. The base or metallic element is put first, thus silver nitrate instead of nitrate of silver. In the chemical formulas only the new notation is given with the atomic weights of elements, as published by Profs. D. Meyer and K. Seubert. The assay method was only introduced for three drugs, cinchona, nux vomica and opium, and their preparations. Few changes have been made in the spelling of medicinal substances, and on the whole the work reflects much credit on the Committee on Revision.

It also advises the general adoption by physicians and pharmacists of the National Formulary issued by the American Pharmaceutical Association, and that the teaching medical and pharmaceutical colleges adopt these works as text-books. H. R. S.

---

### A New Illustrated Dictionary of Medicine, Biography and Collateral Sciences.

Dr. George M. Gould, already well known as the editor of two small Medical Dictionaries, has now about ready an unabridged, exhaustive work of the same class, upon which he and a corps of able assistants have been uninterruptedly engaged for several years.

The feature that will attract immediate attention is the large number of fine illustrations that have been included, many of which—as, for instance, the series of over fifty of the bacteria—have been drawn and engraved especially for the work. Every scientific-minded physician will also be glad to have defined several thousand commonly used terms in biography, chemistry, etc.

The chief points, however, upon which the editor relies for the success of his book is the unique epitomization of old and new knowledge. It contains a far larger number of words than any other one-volume medical lexicon. It is a new book, not a revision of the old volume. The pronunciation, etymology, definition, illustration, and logical groupings of each word are given. There has never been such a gathering of new words from the living literature of the day. It is especially rich in tabular matter, a method of presentation that focuses, as it were, a whole subject so as to be understood at a glance.

The latest methods of spelling certain terms, as adopted by various scientific bodies and authorities, have all been included, as well as those words classed as obsolete by some editors, but still used largely in the literature of to-day, and the omission of which in any work aiming to be complete would make it unreliable as an exhaustive work of reference.

The publishers announce that, notwithstanding the large outlay necessary to its production on such an elaborate plan, the price will be no higher than that of the usual medical text-book.

---

### LIPPINCOTT'S MAGAZINE FOR OCTOBER, 1893.

The complete novel in the October number of *Lippincott's* is "The Hepburn Line," by Mrs. Mary J. Holmes. It is a pleasing tale of an old Kentucky family and a neglected heroine who comes to her own at last.

The eighth in the series of Lippincott's Notable Stories is "A Deed with a Capital D," by Charles M. Skinner. Other short stories are "Poor Yorick,'" by Robert N. Stephens, and "The Pass'n's Grip," by Rosewell Page; both are illustrated.

"Two Belligerent Southrons," by Florence Waller, tells of the bloodless duel between Clay and Randolph, and includes documents never before printed. It is accompanied by portraits, as are also Virginia Butler's account of "An Hour at Sir Frederick Leighton's," and the pair of professional articles, "Necromancy Unveiled" and "Confessions of an Assistant Magician," by Prof. and Mme. A. Herrman.

"Running the Blockade," by Emma Henry Ferguson, is an interesting account of a lady's experience on what was perhaps the last vessel to escape from Wilmington to Bermuda. It is illustrated.

"A Tiger Trapped," an amusing comedietta in one act, by Rosemary Baum, has its scene at Princeton.

W. H. Babcock writes of "Fun in the Poets," and M. Crofton, in "Men of the Day," discusses Henry Labouchere.

The poetry of the number is furnished by Kathleen R. Wheeler, Carrie Blake Morgan, Lucile Rutland, Dr. Titus Munson Coan and Wilbur Larremore.

Eugene Cowles supplies the music of a song, "Once in a Purple Twilight."

